# Pseudoarthrosis of the Femur Secondary to Tuberculosis: A Rare and First Report

**DOI:** 10.7759/cureus.50841

**Published:** 2023-12-20

**Authors:** Rajni Ranjan, Rakesh Kumar, Madhan Jeyaraman, Naveen Jeyaraman, Sankalp Yadav

**Affiliations:** 1 Orthopedics, School of Medical Sciences and Research, Sharda University, Greater Noida, IND; 2 Orthopedics, ACS Medical College and Hospital, Dr. MGR Educational and Research Institute, Chennai, IND; 3 Medicine, Shri Madan Lal Khurana Chest Clinic, New Delhi, IND

**Keywords:** femur, pseudoarthosis, tuberculosis, tb, mycobacterium tuberculosis (mtb)

## Abstract

Tuberculosis poses a major health problem worldwide, and more so in developing countries. Tuberculosis will exist for as long as there are facets of malnutrition, poor sanitation, overcrowding, and immunocompromised populations. We report a rare case of pseudoarthrosis of the femur secondary to tuberculosis. A five-year-old female child presented with swelling, discharging sinuses, and abnormal mobility in the right lower one-third of the thigh secondary to trauma seven months ago. Incision, drainage, and debridement were done, and the obtained pus showed no growth. The sample turned out to be acid-fast bacilli-positive. The patient was on anti-tubercular drugs for six months and had a protective plaster cast for about six weeks, following which knee mobilization was started. During knee mobilization, the patient underwent a forced manipulation of the lower end of the femur, and the radiograph revealed a pathological fracture for which one-and-a-half hip-spica was applied. Further radiographs revealed an un-united fracture after three months despite hip spica application, and a pseudoarthrosis of the right distal femur developed, for which non-vascularized fibular strut grafting for pseudoarthrosis of the distal third of the femur was performed and stabilized with two 2.5 mm-long K-wires supplemented with hip spica for six months. The patient was followed up regularly, and subsequent radiographs showed fibular uptake and resolution of pseudoarthrosis of the femur at the eighth-month follow-up. The patient showed complete resolution of pseudoarthrosis and an excellent functional outcome by the end of the two-year follow-up.

## Introduction

Despite being rare in developed countries, tuberculosis (TB) is still a commonly prevalent disease in developing countries, especially in Asia, Africa, and Europe [1]. Only a small fraction of all TB patients (1-3%) have skeletal TB, which affects the long bones, with most of these patients not having any active lung infection [2]. Due to the scarce presence of these bacteria in bone lesions, bone smears often turn out to be negative, and radiographic findings remain non-specific. This, combined with a low index of clinical suspicion, creates a diagnostic dilemma in such circumstances [1].

Cystic skeletal tuberculosis is an uncommon type of tuberculosis that mainly affects children. It usually involves the metaphysis of the peripheral long bones. Pseudoarthrosis of the femur has only been documented previously as a congenital anomaly associated with constriction bands, associated syndromes such as Ehler-Danlos and neurofibromatosis, or following trauma that is associated with femur neck fractures [3-6]. We are reporting the first instance of tuberculosis-induced pseudoarthrosis of the femur shaft, along with its clinical presentation and management.

## Case presentation

A five-year-old female child presented with swelling, discharging sinuses, and abnormal mobility in the right lower one-third of the thigh. The informant gave a history of injury to the thigh seven months prior, following which the patient developed fluctuant swelling and a high-grade fever for seven days. Incision, drainage, and debridement were done, and the obtained pus showed no growth, which, however, turned out to be acid-fast bacilli (AFB) positive on Ziehl-Neelsen staining. The genotype testing for *Mycobacterium tuberculosis* was confirmed using GeneXpert (Cepheid, Sunnyvale, California, United States), a polymerase chain reaction (PCR), thereby confirming the diagnosis.

**Figure 1:**
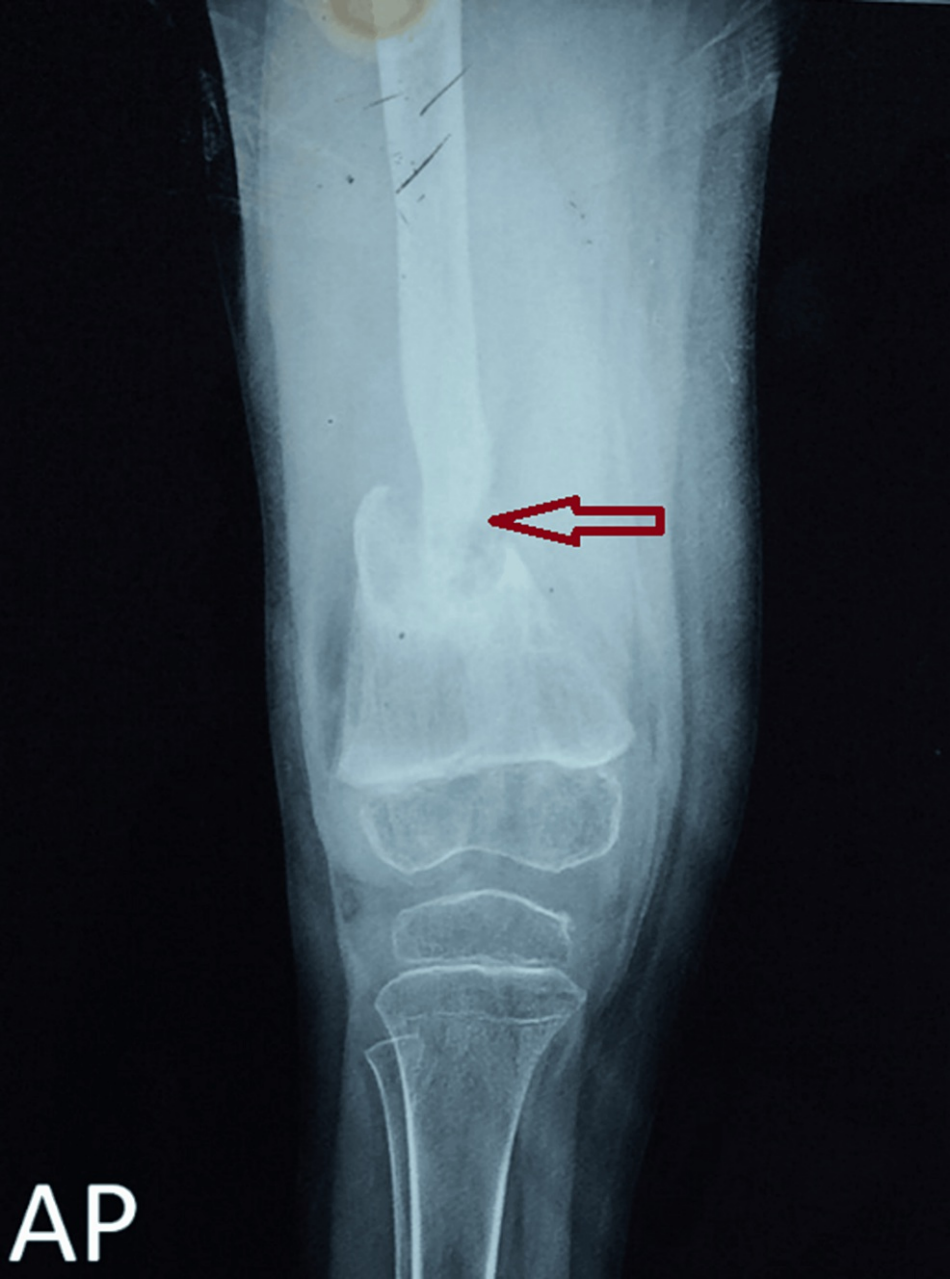
A plain radiograph (AP view) of the right distal femur with the right knee joint showing pathological fracture of the distal one-third of the right femur

The patient was started on anti-tubercular therapy for six months with rifampicin, pyrazinamide, ethambutol, and isoniazid, per the guidelines, and was put on a protective above-knee plaster cast for about six weeks, following which knee mobilization was started. While starting knee mobilization after six weeks, the patient reported a forceful crack at the lower end of the femur during manipulation, which on radiographic evaluation showed the presence of a pathological fracture at the lower-third of the femur, for which one-and-a-half hip-spica was applied (Figure 1).

**Figure 2:**
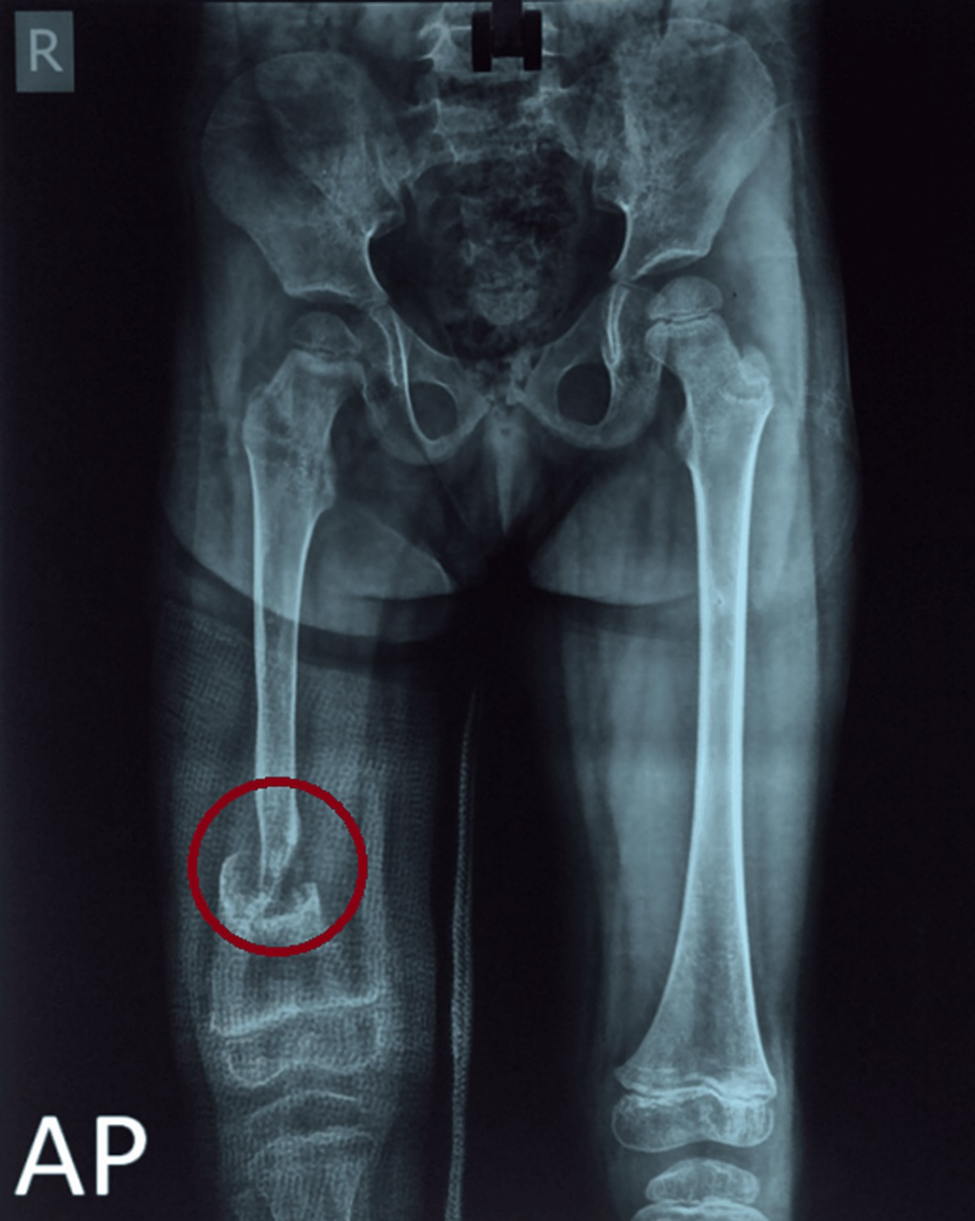
A plain radiograph (AP view) of the pelvis with bilateral hips and a whole-length femur shows pseudoarthrosis of the distal one-third of the right femur

Further radiographs revealed no signs of healing at the end of three months, despite hip spica application, and a pseudoarthrosis of the right distal femur developed (Figure 2).

**Figure 3:**
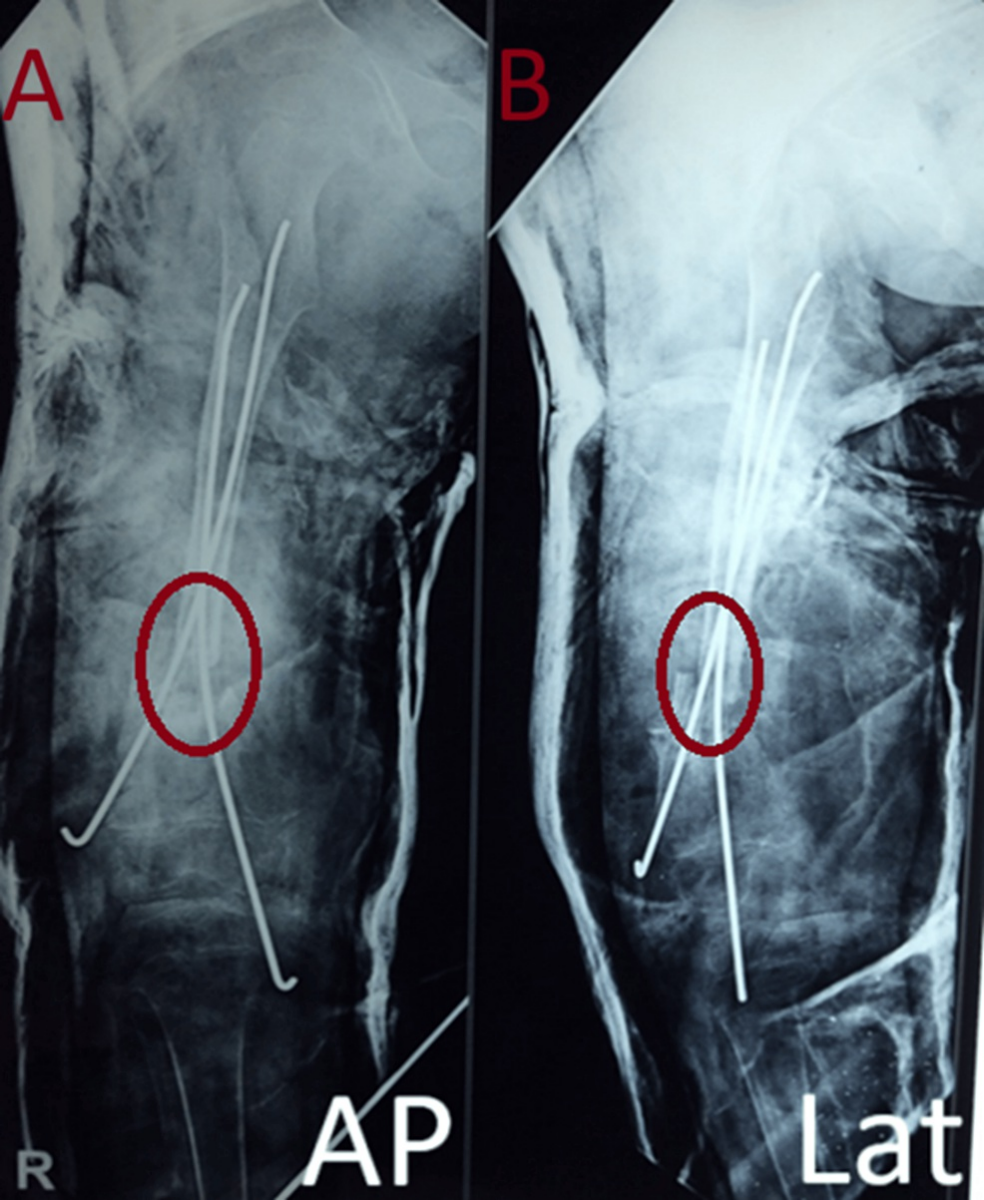
Immediate post-operation radiographs with right femur showing two K-wire fixations with fibular struct grafting at the distal one-third of right femur supplemented with one-and-a-half hip-spica A: AP view, B: Lateral view

The patient further presented with an inability to stand and walk, along with the shortening of the right lower limb with abnormal mobility in both planes at the mid-thigh level. The surgical options for this patient were limited due to the scarcity of bone graft availability from the iliac crest because of the small size of the pelvis. As a result, a non-vascularized fibular strut graft was chosen. After discussing the nature of the illness and surgical options with the informant, consent was obtained for non-vascularized fibular strut grafting augmentation at the pseudoarthrosis site of the distal femur. Bone edges were freshened, the medullary canal was reamed, and the harvested fibular strut graft was impacted into both ends of the femur and stabilized using two 2.5mm K-wires (Figures 3A, 3B).

**Figure 4:**
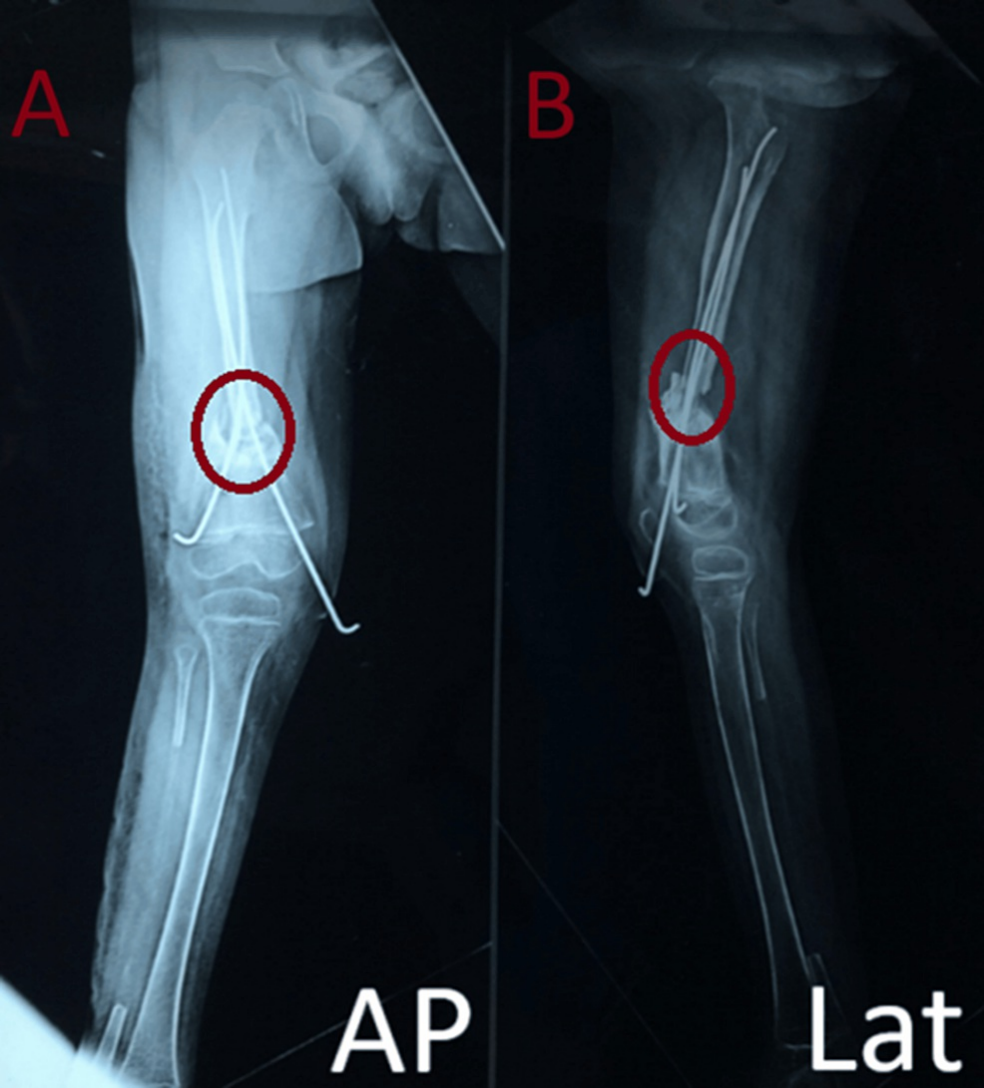
Second-month follow-up radiographs showing progressive signs of fracture union A: AP view, B: Lateral view

**Figure 5:**
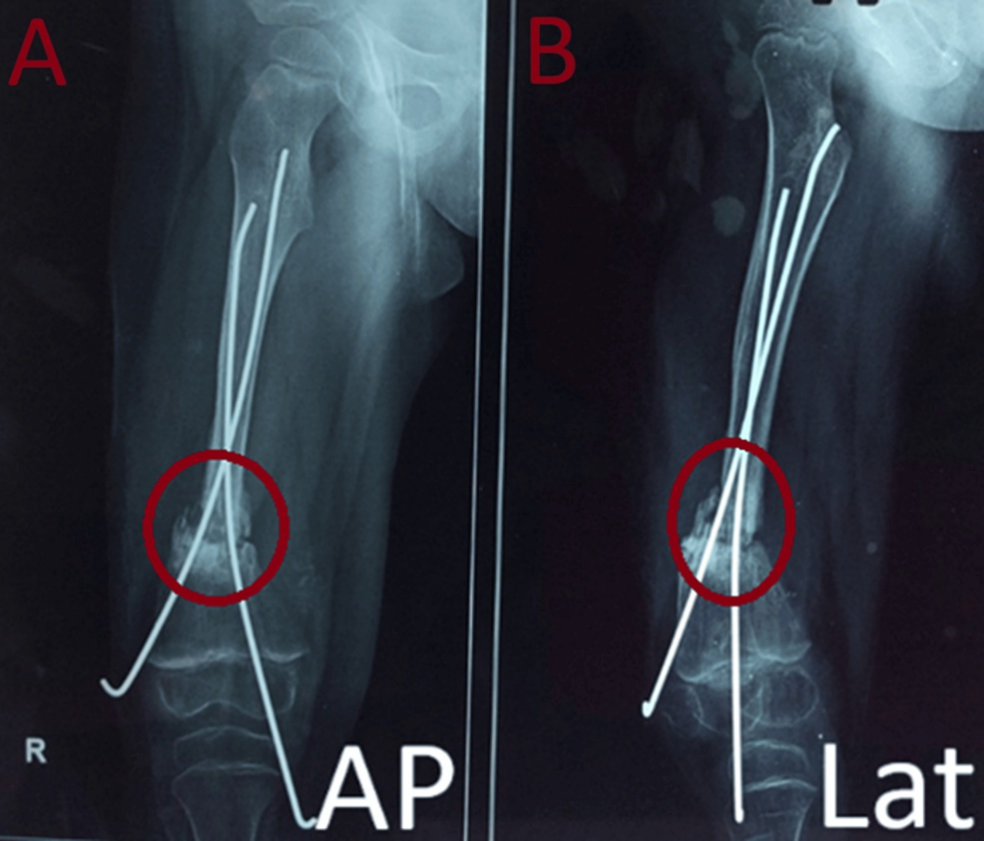
Third-month follow-up radiographs showing progressive signs of fracture union A: AP view, B: Lateral view

**Figure 6:**
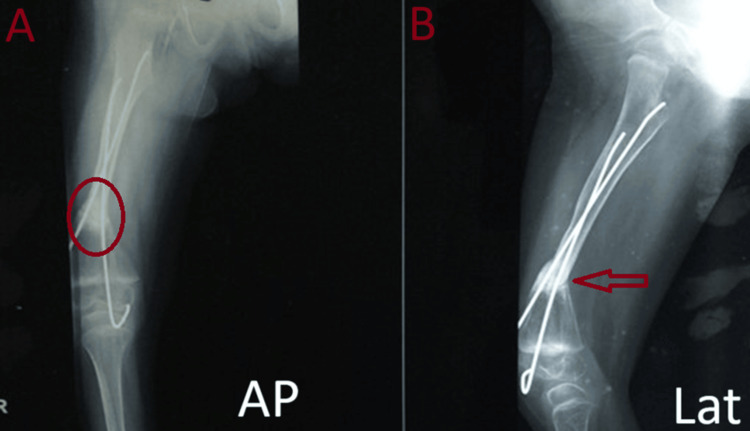
Eighth-month follow-up radiographs showing a progressive sign of fracture union A: AP view, B: Lateral view

This was supplemented with a one-and-a-half hip-spica application for six months. The patient was followed up regularly, and subsequent radiographs revealed gradual fibular uptake and resolution of the femoral pseudoarthrosis by the end of eight months (Figures 4-6).

**Figure 7:**
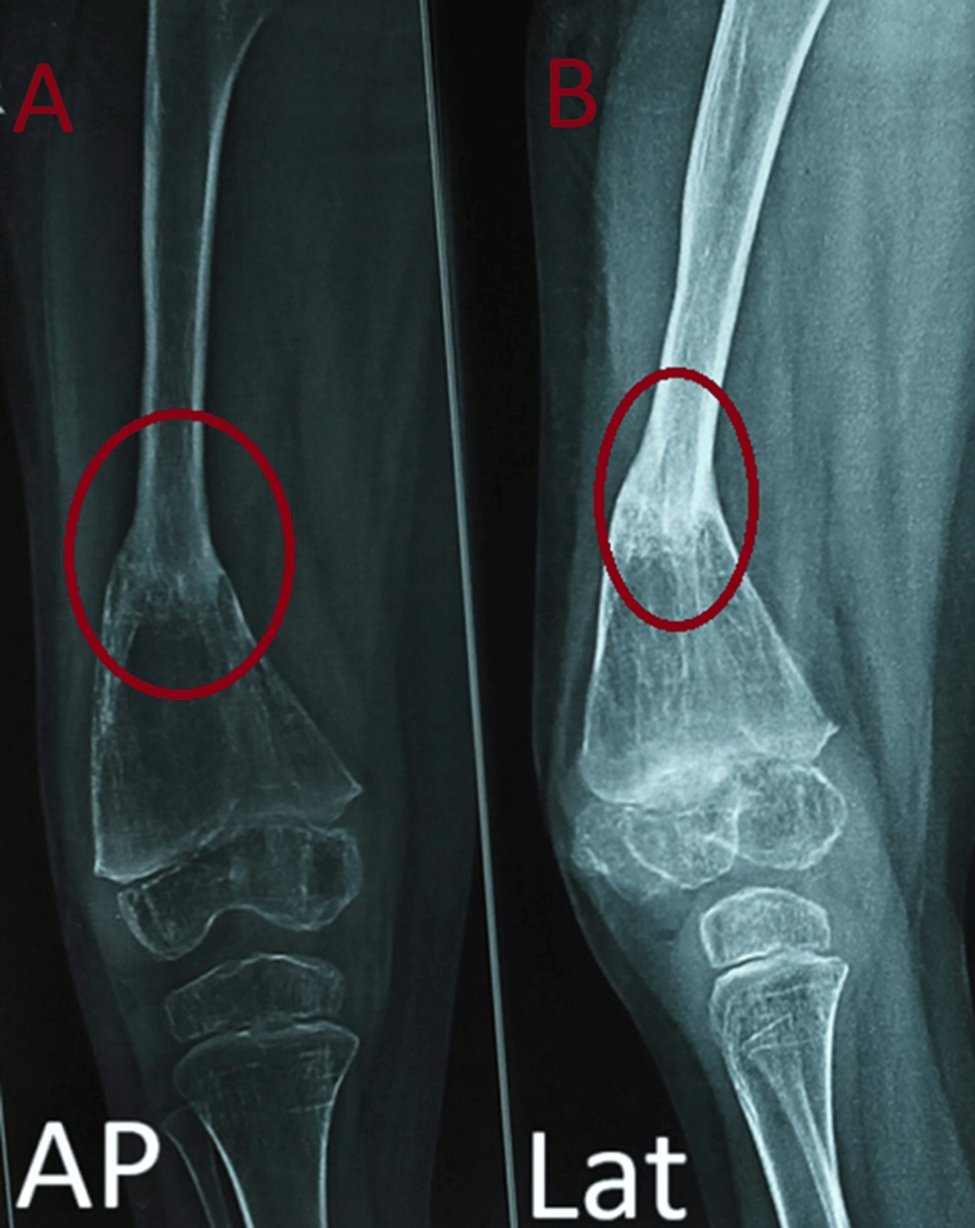
Second-year follow-up radiograph showing satisfactory union at the fracture site A: AP view, B: Lateral view

**Figure 8:**
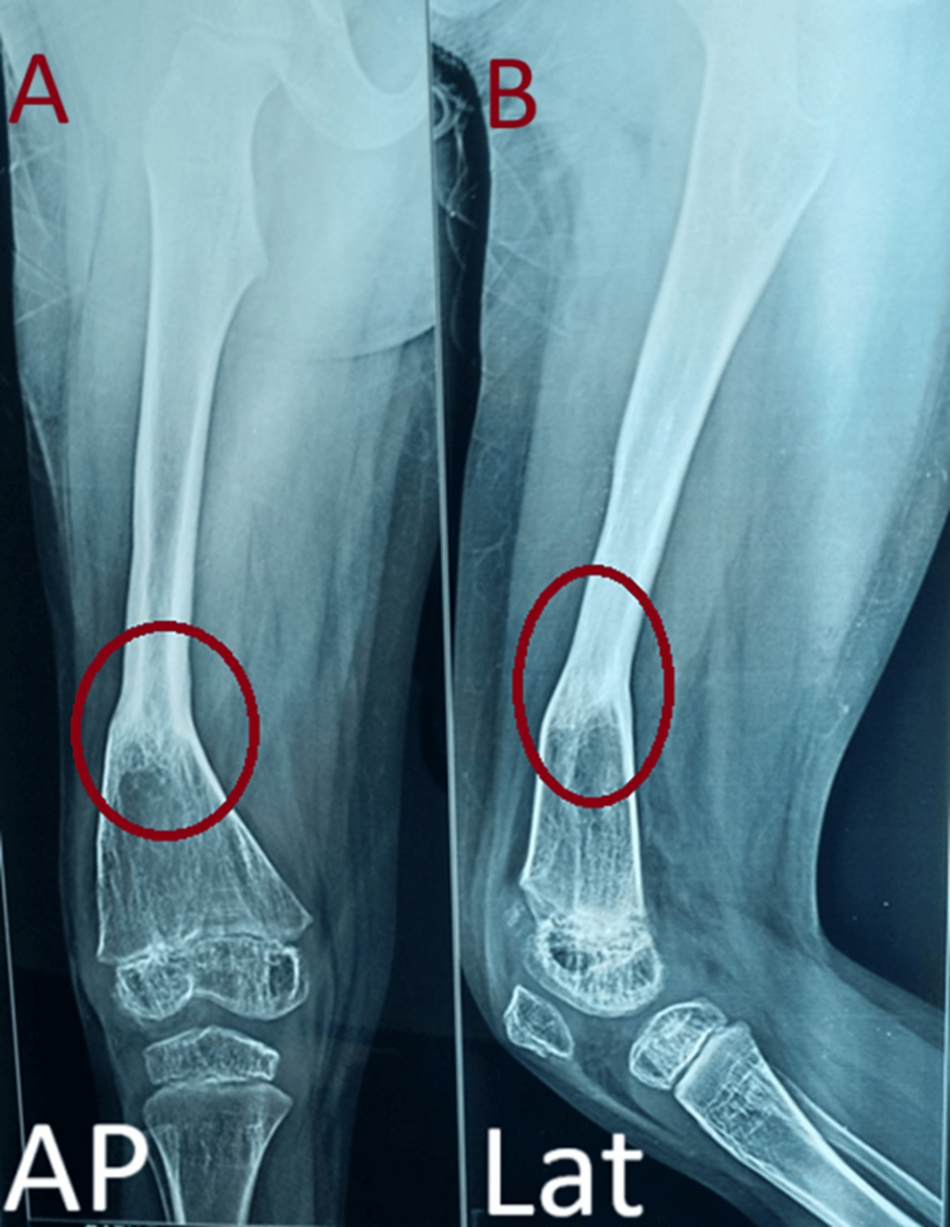
Sixth-year follow-up showing satisfactory union at the fracture site A: AP view, B: Lateral view

The patient showed complete resolution of pseudoarthrosis and excellent functional outcome by the end of the second- and sixth-year follow-ups (Figures 7A, 7B, 8A, 8B).

## Discussion

Skeletal TB can affect any part of the skeleton, among which the spine remains the most common (50%), followed by long bones. Unlike pyogenic osteomyelitis, tuberculosis of the bones in infants and children tends to arise in the vascularized metaphyses, where it causes endarteritis [7-9]. TB of the joints is usually monoarticular, with the knee and hip being the most commonly affected [10,11]. The respiratory system, although the primary route of entry, remains largely inactive. It is hypothesized that tubercular osteomyelitis tends to occur due to lymphohematogenous dissemination to the bone during the initial lung infection, with local reactivation occurring afterward [9,11].

Pseudoarthrosis is generally associated with unsatisfactory outcomes, despite surgical intervention. Song et al. reported the only other case of spontaneous healing of a post-traumatic femur pseudoarthrosis following Ilizarov fixation in a seven-year-old girl. They showed that femur pseudoarthrosis after 4 cm of lengthening showed spontaneous callous formation within four weeks after applying the fixator, and bone union was achieved in four months [12].

Free fibula transfer is a well-documented procedure with good outcomes in children with congenital pseudoarthrosis of the tibia (CPT). Kalra et al. in their study on 26 children with congenital pseudoarthrosis of the tibia showed that a majority of the cases (N-24, 92.6%) achieved primary union with free vascularized fibular transfer, with the majority of the cases showing radiological signs of union within three months (N-20, 76.9%) [13]. Korompilias et al., in their study on vascularized free fibular transfer, also showed a good union within 3.7 months in children with CPT managed using either an external fixator, internal fixation, or intramedullary pins [14]. The main complications associated with a vascularized fibular transfer remain, which include donor site morbidity, insufficient consolidation, limb length discrepancy, and stress fractures, which were not documented in our case report, probably due to the longer period of immobilization [15,16].

This case emphasizes the significance of maintaining a high index of suspicion when evaluating a patient with an unusually destructive bone lesion, especially in a susceptible epidemiologic and clinical setting. It is also important to be familiar with the various radiologic features of TB so that early biopsies and microbiologic testing can be carried out, thereby reducing diagnostic delays. This report also confirms that, along with adequate anti-tubercular treatment and minimal surgical intervention, good bone union can be expected in children.

## Conclusions

The management of tubercular pseudoarthrosis of the femur, a rare condition, hinges on early recognition and a high index of clinical suspicion, particularly in tuberculosis-endemic areas. Effective treatment necessitates a dual approach: comprehensive anti-tubercular therapy to address the infection and surgical intervention, notably the use of a non-vascularized fibular graft, to promote bone union. This strategy, requiring meticulous postoperative care and regular follow-up, can lead to favorable outcomes in terms of bone healing and functional recovery. This case highlights the importance of integrating medical and surgical modalities in the management of complex tubercular skeletal conditions.
